# Supplementary material for: Investigating self-recognition in bonobos: mirror exposure reduces looking time to self but not unfamiliar conspecifics
Source: PeerJ. 2020 Aug 28;8:e9685. doi: 10.7717/peerj.9685 (PMC7457926; doi:10.7717/peerj.9685)
Supplement: Table S1 [file peerj-08-9685-s001.docx]

Table S1. Experimental conditions, definition and device used for each experiment.

| Condition | Stimulus description | Device | Part |
| --- | --- | --- | --- |
| *C self* | Contingent video image of self with no direct eye contact | iPad | 1 |
| *Mirror* | Contingent image of self with direct eye contact | Mirror | 1 |
| *NC self* | Non-contingent video footage of self | iPad | 1 & 2 |
| *Known* | Video footage of known group member | iPad | 1 |
| *Unknown* | Video footage of unknown conspecific | iPad | 1 & 2 |
