# Supplementary material for: Investigating self-recognition in bonobos: mirror exposure reduces looking time to self but not unfamiliar conspecifics
Source: PeerJ. 2020 Aug 28;8:e9685. doi: 10.7717/peerj.9685 (PMC7457926; doi:10.7717/peerj.9685)
Supplement: Table S2 [file peerj-08-9685-s002.docx]

Table S2. Experimental timeline - Condition presentation sequence, before (part 1) and after (part 2) prolonged mirror exposure.

|  | Part 1 | | | | | | | | | | | | | | |  |  |  |  |  |  |  |  |  |  | Part 2 | | | | | | | | |
| --- | --- | --- | --- | --- | --- | --- | --- | --- | --- | --- | --- | --- | --- | --- | --- | --- | --- | --- | --- | --- | --- | --- | --- | --- | --- | --- | --- | --- | --- | --- | --- | --- | --- | --- |
|  | Before mirror exposure | | | | | | | | | | | | | | |  | | | | | | | | | | After mirror exposure | | | | | | | | |
| *C self* |  | |  |  |  |  |  |  |  |  |  |  |  |  |  |  |  |  |  |  |  |  |  |  |  |  |  |  |  |  |  |  |  |  |
| *mirror* |  |  |  |  |  |  | |  |  |  |  |  |  |  |  |  |  |  |  |  |  |  |  |  |  |  |  |  |  |  |  |  |  |  |
| *NC self* |  |  |  |  |  |  |  | * |  |  |  |  |  |  |  |  |  |  |  |  |  |  |  |  |  | * | |  |  |  |  |  |  |  |
| *known* |  |  |  |  |  |  |  |  |  |  |  |  |  |  |  |  |  |  |  |  |  |  |  |  |  |  |  |  |  |  |  |  |  |  |
| *unknown* |  |  |  |  |  |  |  |  |  |  |  |  |  | * | |  |  |  |  |  |  |  |  |  |  |  |  |  |  |  |  | * | |  |
| mirror experience status | Naive | | | | | | | | | | | | | | | | | | | *Ad libitum* mirror exposure | | | | | | Experienced | | | | | | | | |
| period | JAN | | FEB | | MAR | | APR | | MAY | | JUN | | JUL | | AUG | | // | | NOV | | DEC | | JAN | | FEB | | MAR | | APR | | MAY | | JUN | |
|  | 2014 | | | | | | | | | | | | | | | |  | | 2015 | | | | 2016 | | | | | | | | | | | |

* For each subject the time-lapse between '*NC self*' presentation before and after mirror exposure is identical to that between '*unknown*' before and after.
